# Phylogenetic Analysis and Rapid Identification of the Whitefly, *Bemisia afer*, in China

**DOI:** 10.1673/031.010.8601

**Published:** 2010-07-01

**Authors:** Dong Chu, Guoxia Liu, Fanghao Wan, Yunli Tao, Ray J Gill

**Affiliations:** ^1^ High-tech Research Center, Shandong Academy of Agricultural Sciences, Jinan 250100, Shandong, China; ^2^ State Key Laboratory for Biology of Plant Diseases and Insect Pests, Institute of Plant Protection (South Campus), Chinese Academy of Agricultural Sciences, Beijing, 100081, China; ^3^ Plant Pest Diagnostic Center, 3294 Meadowview Road, Sacramento, California, 95832 USA

**Keywords:** mitochondrial cytochrome oxidase I, molecular markers

## Abstract

The phylogenetic relationship between the whitefly *Bemisia afer* (Priesner & Hosny) (Hemiptera: Aleyrodidae) from China and other populations among the world were analyzed based on the mitochondrial cytochrome oxidase I (mtCOI) gene. Phylogenetic analysis of mtCOI sequences and those of reference *B. afer* sequences showed that the populations of the species could be separated into 5 clades (I–V). There were at least two clades of the species from China (IV and V). These data suggested that *B. afer* might be a species complex. The Chinese *B. afer* populations were most divergent with *B. afer* from the United Kingdom and African countries. The distance between the Chinese *B. afer* (IV and V) and clades I, II, and III is more than 32%, while the distance among clades I, II, III is lower than 7.7%. A new set of primers specific to *B. afer* was designed to amplify a region of approximately 400 bp to discriminate *B. afer* from other *Bemisia* species in China based on mtCOI sequences.

## Introduction

During recent years, the outbreak of *Bemisia tabaci* (Priesner & Hosny) (Hemiptera: Aleyrodidae) and the serious damage caused by this whitefly pest in many countries have required researchers to study the biological and ecological characteristics of and effective control strategies against it (Brown et al. 2000; Brown 2007; De Barro et al. 2000, 2003). The identification of species in the genus *Bemisia* is the basis of this research, but the taxonomy of whiteflies has long been problematic because of similarities in the morphology of pupae and adults. Pupae of *Bemisia* species exhibit phenotypic variation in response to differences in leaf surface topology and to environmental and physical factors (Maruthi et al. 2007).

In China, *B. tabaci* became the major pest of the fiber crops, ornamental plants and vegetables (Chu et al. 2006, 2007, 2008). During field research, a *Bemisia* species, which was difficult to distinguish from *B. tabaci,* was discovered on *Broussonetia papyrifera* (Linn.) Vent. (Urticales: Moraceae). Based on the morphological characteristics of pupae and adults, the species was identified as *B. afer.* As *B. tabaci* has several close relatives and numerous biotypes, *B. afer* also is likely to have many forms and cryptic species. Earlier studies indicated that *B. afer* exhibits much greater morphological variation than does *B. tabaci* and its variants (Anderson et al. 2001; Maruthi et al. 2007). The Chinese *B. afer* has slightly different morphology compared to *B. afer* from other geographical locations, and it is expected that these differences would be reflected at the molecular level.

The mitochondrial cytochrome oxidase I (mtCOI) gene has been used extensively as a molecular marker to identify *B. tabaci* variants that exhibit rich biological differences (Frohlich et al. 1999; Hsieh et al. 2007) but lack distinguishing morphological features. Previous studies have shown that mtCOI sequences also are informative for identifying *B. afer* variants, which lack distinguishing morphological features (Maruthi et al. 2007). In this study, the mtCOI gene of *B. afer* was sequenced using the primer set (C1-J-2195 and L2-N-3014) that has been used extensively on *B. tabaci,* and the fragments were also sequenced. The phylogenetic relationships among the world populations were analyzed. The infection status of an endosymbiont *Wolbachia* of Chinese *B. afer* was studied because it often causes reproductive incompatibilities between infected and uninfected hosts, which can affect the divergence of mtDNA and can facilitate or even cause host speciation (Werren 1997; Ballard et al. 2004; Shoemaker et al. 2004). Finally, the specific primers to Chinese *B. afer* were designed based on the sequences of the mtCOI gene of Chinese *B. afer* and *B. tabaci* biotypes B and Q.

The objectives of the paper are: 1) to further analyze the phylogenetic relationships, based on the mitochondria COI gene, between Chinese populations of *B. afer* on *B. papyrifera* with other populations of *B. afer* from the United Kingdom and African countries and to discuss the relationship between the divergence of *B. afer* and the endosymbiont, *Wolbachia;* 2) to develop a rapid molecular marker based on the mtCOI gene to distinguish *B. afer* from *B. tabaci* biotypes B and Q, which are the predominate biotypes in China, especially the biotypes in the Shandong province. The aim is to contribute to the understanding of the systematic status of *B. afer* populations in China and the genetic differentiation of *B. afer* worldwide.

## Materials and Methods

### Collection of the samples and species identification

During 2006 and 2007, pupae and adults of *Bemisia* species on *B. papyrifera* were collected alive and placed singly into tubes containing 95% ethanol. The species were identified based on the pupae and adults.

### DNA extraction and PCR

Genomic DNA was extracted from individual adults according to the method described previously by Frohlich et al. (1999). Polymerase chain reaction (PCR) was employed to amplify fragments of the *B. afer* mitochondrial COI gene (800–820 bp), using parameters and PCR primers (C1-J-2195 and L2-N-3014) as described by Frohlich et al. (1999).

PCR assays were conducted using 2 µl of each template DNA in a total reaction volume of 25 µl. PCR conditions follow Frohlich et al. (1999), with 1 unit of Taq DNA polymerase. PCR products were separated on 1.0% agarose gel. The bands were visualized by ethidium bromide staining and viewed with a UV light source.

### Cytochrome oxidase I sequencing and phylogenetic analysis

PCR products were purified using an EZ Spin Column DNA Gel Extraction Kit (Sangon Technology Company, www.sangon.com/index.htm) according to the manufacturer's instructions. The DNA sequence for each PCR product was determined from the 5'end at the Sangon Technology Company. The mtCOI sequences determined were deposited in GenBank.

Phylogenetic analysis included all available mtCOI sequences from GenBank and sequences from this study, with the sequences of *Bemisia tabaci* (mainly including the indigenous biotypes from China), *B. tuberculata, B. berbericola, Trialeurodes vaporariorum,* and *Trialeurodes abutilonea* as the outgroup (Table 1). The mtCOI sequences were aligned using the CLUSTAL W algorithm (Thompson et al. 1994). The aligned mtCOI sequences of ∼ 600 bp are presented. Distances based on the mtCOI sequences of ∼600 bp were calculated based on the Kimura 2-parameter model using MEGA 4.1 (Tamura et al. 2007). The ME (Molecular Evolution) and MP (Maximum Parsimony) algorithms available in MEGA 4.1 were used to infer phylogenetic relationships from the sequences. One thousand Bootstrap replicates were performed for each analysis.

On the basis of the results of phylogenetic analysis, the *B. afer* specimens were separated into five subclades. The sequences in the subclades were selected to further calculate distances within and between group average calculations using MEGA 4.1.

### *Wolbachia* detection of Chinese *B. afer*

All *B. afer* were also screened for *Wolbachia* infection by PCR, employing the primers wsp81F and wsp691R (Zhou et al. 1998), which amplify part of the *Wolbachia* surface protein gene (*wsp*). The study included 15 Chinese *B. afer* individuals, and the PCR was repeated three times.

### Development of the specific diagnostic test

Experiments showed that the primers C1-J-2195 and L2-N-3014 did not always amplify products of the expected size, although the DNA was useful. A set of primers specific to the *B. afer* mtCOI gene was designed, which included the newly designed forward primer Bafer-J2 (5′-GTTAGTTTTGGGGATTAGTC-3′) by aligning in CLUSTAL W (Thompson et al. 1994) and the reverse primer L2-N-3014 (5′-TCCAATGCACTAATCTGCCATATTA-3′). The other whitefly species (Table 2) were used in PCR reactions to test the specificity of primers to *B. afer.* The PCR reaction mix followed the method previously described by Frohlich et al. (1999). Reactions used the following PCR program: 94° C for 2 min; followed by 30 cycles of 94° C for 1 min, 54° C for 1 min, and 72° C for 1 min; and ending with 72° C for 5 min. PCR products were separated on 1.0% agarose gel. The bands were visualized by ethidium bromide staining and viewed with a UV light source.

**Table 1.**
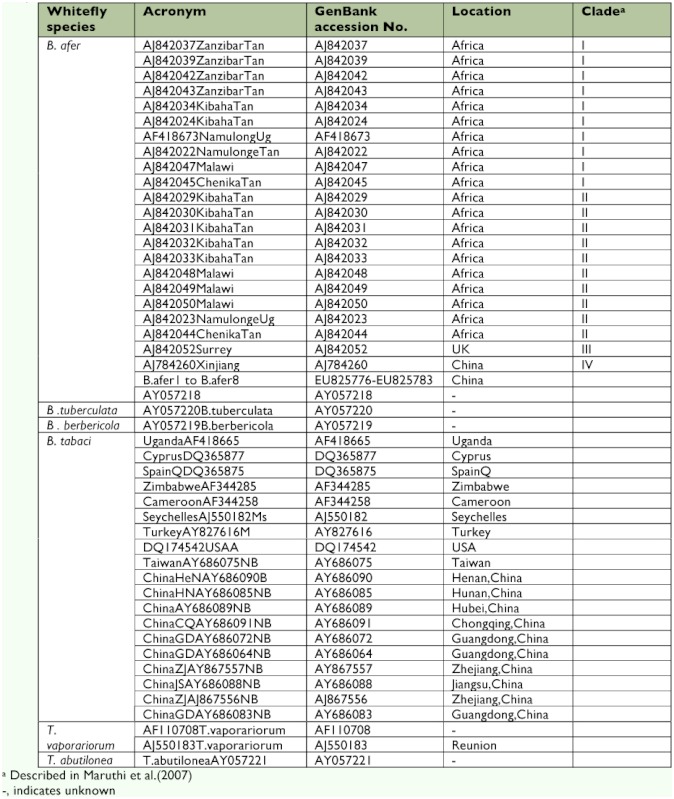
Detail of *Bemisia afer* sample and other whiteflies species collections and their mtCOI gene sequences used in the study.

## Results

### Morphology of *B. afer*

Adults of New World specimens of *B. afer* have the upper and lower eyes completely separate in both sexes, as contrasted with *B. tabaci* adults,which have one ommatidium connection. Some *B. afer* from the Macaronesian Islands have the male eyes connected by one ommatidium but separated in the female, while others from different hosts have both eyes separate as in the North American forms (RJ Gill, unpublished data). In the specimens for this study, the female had a one ommatidium connection, while the male had both eyes connected positively with one ommatidium, but also another ommatidium almost but not touching. In the adults, there were clear morphological differences.

### Phylogenetic analysis of *B.afer*

**Figure 1.**
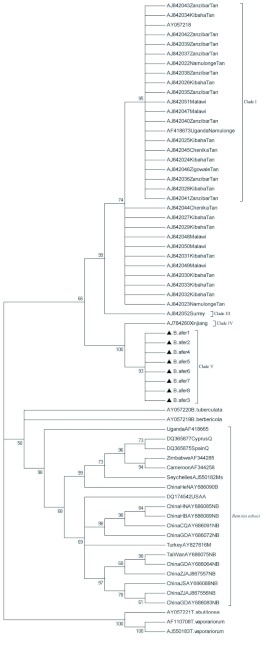
ME (Minimum Evolution) tree based on ∼600-bp fragment of the mtCOI sequences. Numbers at nodes indicate bootstrap scores using 1000 replicates. Abbreviations are as described in Table 1. 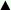 indicates sequences obtained in this study. High quality figures are available online.

A total of 8 *B. afer* mtCOI gene sequences of about 600 bases were obtained from populations in Shandong, China during 2006 and 2007. The GenBank accession numbers are EU825776 to EU825783. The phylogenetic tree generated with the Minimum Evolution (ME) method is shown in Figure 1. The tree generated with MP (Maximum Parsimony) method (not shown) is similar to Figure 1. The tree that was generated by heuristic research had 65% confidence level. Based on the trees, *B. afer* could be separated into 5 clades. The populations from Xinjiang, China (AJ784260Xinjiang) and Shandong, China (Bafer 1–Bafer8) were grouped in clade IV and V, respectively. The Chinese *B. afer* populations (clade IV and V) were most divergent with clades I, II, and III.

**Table 2.**
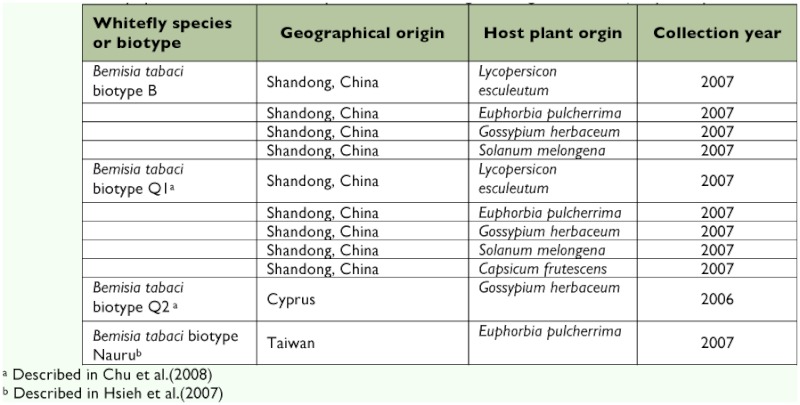
Whitefly species used in the PCR amplification of mtCOI gene using the *Bemisia afer* specific primers

### Genetic differentiation of the *B.afer* in China and *Wolbachia* infection

Average distances within and between clades based on mtCOI of whiteflies are summarized in Table 3. The Chinese *B. afer* populations were most divergent with *B.afer* from the United Kingdom and African countries. The distance between the Chinese *B. afer* (clade IV and V) and clades I, II, and III was more than 32%), while the distance among clades I, II, and III were lower than 7.7%. No *Wolbachia* could be detected in Chinese populations of *B.afer* (clade V).

### Specific primers for the *B. afer* in China

**Figure 2.**
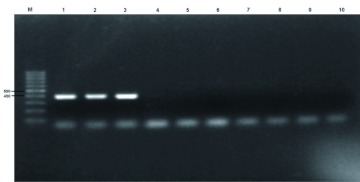
PCR products generated using the *Bemisia afer* specific primers (Bafer-J2 and L2-N-3014) (lanes 1–3); *Bemisia tabaci* biotype B (lanes 4–5), biotype Q1 (lanes 6–7), biotype Q2 (lane 8–9), biotype Naura (lane 10). M: 100 bp molecular weight marker, the sizes of which are shown on the left. High quality figures are available online.

Figure 2 shows the PCR products generated from the DNA of several whitefly species using Bafer-J2 and L2-N-3014 primers. A band of approx 400 bp was obtained from DNA of *B. afer* from Shandong, China. No specific PCR products were obtained from DNA of *B. tabaci* biotype B, Q1, Q2, or Nauru.

**Table 3.**
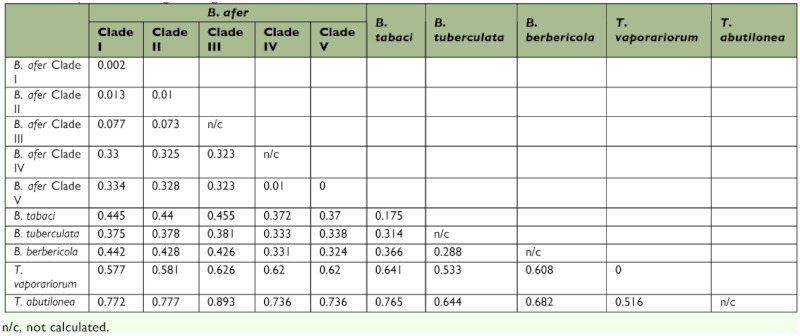
Average distance within and between clades of whiteflies based on mtCOI. The genetic distance among the haplotypes within each clade is presented along the diagonal.

## Discussion

The phylogenetic analysis based on the mtCOI sequences suggested that the *B. afer* is a species complex that includes many genetically divergent clades. The result based on the molecular marker is consistent with the analysis based on the morphological characteristics.

This study revealed the presence of at least 5 clades in *B. afer* worldwide. The endosymbiont *Wolbachia* was not detected in *B. afer* and may not have affected the evolution of the Chinese *B. afer* (clade V). Studies with higher sample sizes are required and detailed examinations of morphological and molecular characters are necessary to understand the *B. afer* species complex.

In China, there are at least two clades of *B. afer* based on the mtCOI sequences. Although this study confirmed the presence of *B. afer* in China, the biology of the Chinese species, including host ranges, is still unknown and should be further studied. This study shows that a simple, PCR-based technique is sufficient for the reliable identification of *B. afer* using a new primer pair designed to amplify a portion of the mtCOI gene, which has been shown to be specific to the Chinese *B. afer* (clade V).

## Abbreviations

mtCOI: mitochondrial cytochrome oxidase I gene
